# A Fresh Look at the Origin of *Plasmodium falciparum*, the Most Malignant Malaria Agent

**DOI:** 10.1371/journal.ppat.1001283

**Published:** 2011-02-24

**Authors:** Franck Prugnolle, Patrick Durand, Benjamin Ollomo, Linda Duval, Frédéric Ariey, Céline Arnathau, Jean-Paul Gonzalez, Eric Leroy, François Renaud

**Affiliations:** 1 Laboratoire MIVEGEC (UM1-CNRS-IRD), Montpellier, France; 2 Unité de Parasitologie Médicale, Centre International de Recherches Médicales de Franceville, Franceville, Gabon; 3 Unité de Recherche en Ecologie de la Santé, Centre International de Recherches Médicales de Franceville, Franceville, Gabon; 4 Unité des Maladies Virales Emergentes, Centre International de Recherches Médicales de Franceville, Franceville, Gabon; University of California San Diego, United States of America

## Abstract

From which host did the most malignant human malaria come: birds, primates, or rodents? When did the transfer occur? Over the last half century, these have been some of the questions up for debate about the origin of *Plasmodium falciparum*, the most common and deadliest human malaria parasite, which is responsible for at least one million deaths every year. Recent findings bring elements in favor of a transfer from great apes, but are these evidences really solid? What are the grey areas that remain to be clarified? Here, we examine in depth these new elements and discuss how they modify our perception of the origin and evolution of *P. falciparum*. We also discuss the perspectives these new discoveries open.

## Introduction

In the genus *Plasmodium*, four species are traditionally regarded as human parasites: *Plasmodium malariae*, *Plasmodium ovale*, *Plasmodium vivax*, and *Plasmodium falciparum*. These species are remotely related to each other, suggesting that adaptation to humans has occurred several times independently during the history of the genus. It is still unclear, however, when these associations began and from where they came [1]. The origin of *P. falciparum*, in particular, continues to be a highly debated topic.

Early molecular phylogenetic studies on the genus *Plasmodium* showed that *P. falciparum* clustered with two avian parasites rather than with those infecting mammals, thus suggesting that *P. falciparum* was the result of a transfer from birds to humans [2], [3]. According to the authors, this transfer took place at the beginning of agricultural development, when the human habitat was settled about 10,000 years ago. This result was quickly questioned, though, due to the small number of ingroup taxa considered for the phylogenetic analyses and the use of 18S rDNA sequences, which have proved their weakness in studies on Haemosporidia phylogeny (e.g., [4]).

Subsequent analyses demonstrated that the closest sister taxon of *P. falciparum* was *Plasmodium reichenowi*, a parasite isolated from a chimpanzee. Escalante and Ayala [5] suggested that these two parasites diverged at the time of the divergence between humans and chimpanzees. According to their results, *P. falciparum* did not directly originate from an avian malarial parasite. Nevertheless, the *P. falciparum/P. reichenowi* pair still was considered as a sister lineage of the parasites from birds and lizards [5].

The results of subsequent studies were contradictory. Some of them concluded that the *P. falciparum/P. reichenowi* clade was included in the mammalian group, being closer to the rodent or the primate *Plasmodium* [6]–[8]; others reached the opposite conclusion by showing that this group clustered more closely with parasites form birds [9]–[16]. Part of the confusion concerning the origin of *P. falciparum* arose because of biases in the representation of certain taxa, the small number of loci analysed and/or improper rooting. In 2008, the study of Martinesen et al. [4] did not suffer from these shortcomings and quite conclusively placed *P. falciparum* basal to all other mammalian malaria parasites. However, the origin of *P. falciparum* within this group of pathogens still remained unknown. Finally, it is only very recently, by adding more taxa from primates (in particular great apes), that the origin of *P. falciparum* was firmly established.

### Great Apes Are Hosts of Higher *Plasmodium* Diversity Than Previously Thought

In one year, the known diversity of *Plasmodium* species infecting great apes and belonging to the *P. falciparum* lineage has burst. Until May 2009, only one species, *P. reichenowi*, was known to be phylogenetically a sister lineage of *P. falciparum*. *P. reichenowi* was isolated from a chimpanzee that was captured near Lake Edwards (Democratic Republic of Congo) [17], likely belonging to the *Pan troglodytes schweinfurthii* subspecies. Until recently, this isolate was the only great ape parasite genetically characterized [18].

In May 2009, Ollomo et al. [19] published the complete mitochondrial genome of a new, not yet described species of *Plasmodium* that circulates in great apes and belongs to the *P. falciparum*/*P. reichenowi* lineage. This species was discovered in two wild-borne chimpanzees kept as pets in villages of Gabon and was called *Plasmodium gaboni* in reference to the country where it was found. Based on the hypothesis that *P. falciparum* and *P. reichenowi* diverged about 6 million years (MY) ago, the authors proposed that *P. gaboni* diverged from the *P. falciparum*/*P. reichenowi* lineage about 21 MY ago, leading them to conclude that the ancestor of this African great apes/human parasite clade (also known as the *Laverania* subgenus, [20]) could have been already present in hominid ancestors [19].

Three months later, Rich and colleagues [21] described the diversity of *Plasmodium* species that circulate among wild and wild-borne chimpanzees from Ivory Coast and Cameroon. From blood samples, the authors identified, using partial sequences of the *Cytochrome B* (*CytB*) gene but also some apicoplastic and nuclear sequences, eight *Plasmodium* isolates that they described as *P. reichenowi*. However, the *CytB* sequences showed a high polymorphism and some of them were actually very close genetically to the previously described sequences of *P. gaboni* [21]. The phylogenetic analysis carried out by the authors suggests that *P. falciparum* arose from a recent transfer from chimpanzees to humans that may have occurred as early as between 5,000 to 50,000 years ago. This finding challenges the conventional belief that *P. falciparum* and *P. reichenowi* diverged at the same time as their respective hosts (human and chimpanzee) between 4 and 7 MY ago.

In January 2010, Prugnolle and colleagues [22] published a paper in which, using new non-invasive methods based on the use of great apes fecal samples, they described the diversity of *Plasmodium* species that circulate in wild West African chimpanzees and also, for the first time, in gorillas (subspecies *Gorilla gorilla gorilla* and *G. gorilla dielhi*). Their study confirmed the presence of *P. gaboni* and *P. reichenowi* in wild chimpanzees (subspecies *P. troglodytes troglodytes* and *P. troglodytes vellerosus*) and reported the existence of still unknown *Plasmodium* genetic lineages in wild gorillas. One clusters with *P. reichenowi* and *P. falciparum* (they called this phylogenetic lineage *P. GorB*) and the other is a sister lineage of *P. gaboni* (they called it *P. GorA*). Their results confirm that chimpanzees are infected by a large diversity of *Plasmodium* species from which *P. falciparum* seems to have originated. In addition, the authors identified *P. falciparum* in wild gorillas, an unexpected result since *P. falciparum* is considered as strictly specific to humans. They did not find any genetic differences between the *P. falciparum* circulating in humans and in gorillas based on the partial *CytB* sequence studied, thus suggesting the possibility of a recent transfer from humans to primates.

In February 2010, Krief and collaborators [23] confirmed the large diversity of *Plasmodium* species infecting great apes from Central Africa. The originality of their work lies in the presence of isolates from chimpanzees from East Africa (subspecies *P. t,. schweinfurthii*) and from bonobos. By sequencing the whole *Plasmodium* mitochondrial genome (as well as some apicoplastic and nuclear genes), they identified two distinct parasite lineages in chimpanzees. One is located at the root between *P. falciparum* and *P. reichenowi* and was called *Plasmodium billcollinsi*. The other one was named *Plasmodium billbrayi* and is phylogenetically very close to *P. gaboni*. Interestingly, they also discovered *P. falciparum* in bonobos and found that the genetic diversity of its mitochondrial genome was higher than the overall diversity found in human *P. falciparum*, thus suggesting that *P. falciparum* might have originated from bonobos and was transferred to humans as early as 30,000 years ago. This interpretation challenges the hypothesis by Rich et al. [21] and Prugnolle et al. [22], according to which *P. falciparum* likely originated from a transfer from chimpanzees.

In May 2010, Duval and colleagues [24] investigated the diversity of *Plasmodium* species in chimpanzees and gorillas from Cameroon by sequencing two mitochondrial genes (*CytB* and *Cox1*). Their study confirmed the existence of the lineage *P. GorB* in western lowland gorillas and of *P. gaboni* and *P. reichenowi* in chimpanzees. They also confirmed the presence of *P. falciparum*–related parasites in gorillas and demonstrated, for the first time, the presence of *P. falciparum* in blood samples from two different chimpanzee subspecies (*P. t. vellerosus* and *P. t. troglodytes*) from sanctuaries.

Finally, very recently, in September 2010, Liu et al. [25] published a study on the diversity of *Plasmodium* species in African great apes based on a very large collection of fecal samples from three subspecies of chimpanzees (*P. t. troglodytes*, *P. troglodytes ellioti* [also known as *P. t. vellerosus*], and *P. t. schweinfurthii*), bonobos, and two subspecies of gorillas (subspecies *G. gorilla gorilla* and *G. gorilla graueri*), and a method of single template amplification allowing them to sequence mitochondrial, apicoplastic, and nuclear genes of *Plasmodium* isolates from mixed infections. From their results, they conclude the existence of six *Plasmodium* species belonging to the *Laverania* subgenus, three in chimpanzees (they called them C1–C3) and three in gorillas (referred to as G1–G3). All these species were previously described, but by the greater depth of sampling, this study gives a more definitive picture of the diversity of the *Laverania* species infecting great apes. In particular, this study confirms the existence of a large diversity of *P. falciparum*–related parasites in gorillas but does not find any in natural populations of chimpanzees or bonobos. This latter finding suggests a likely gorilla origin for human *P. falciparum*, in opposition to all theories previously proposed.

### Update on the Diversity of *Plasmodium* Circulating in African Great Apes…

**Figure 1:**
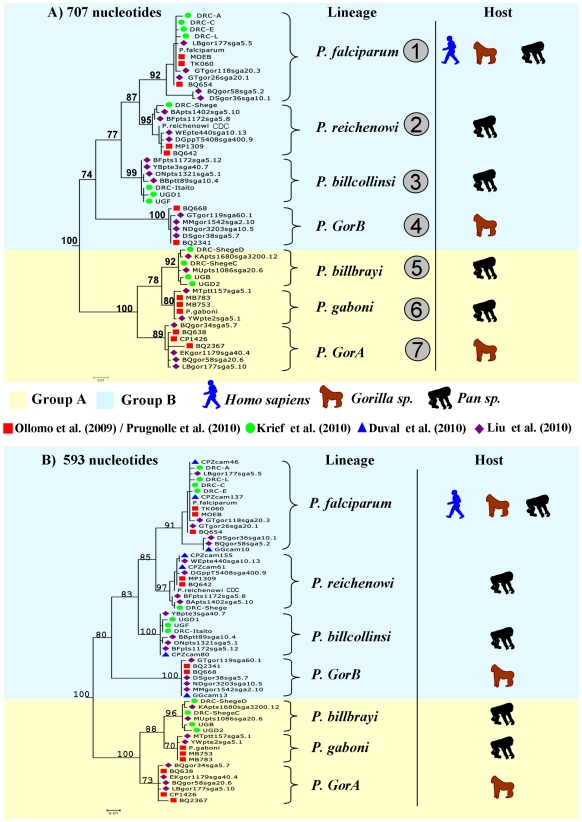
Phylogeny of the *Laverania* subgenus. This phylogeny is based on partial *CytochromeB* sequences and including strains isolated and characterized in (A) Ollomo et al. [19], Prugnolle et al. [22], Krief et al. [23], and Liu et al. [25], and in (B) Ollomo et al. [19], Prugnolle et al. [22], Krief et al. [23], Duval et al. [24], and Liu et al. [25]. The phylogenies were produced using a maximum likelihood approach and robustness was tested using 100 bootstraps. Names of the lineages were given following their first denomination (see Table 1) except for *P. billcollinsi*, which was first named by Rich et al. [21] as *P. reichenowi*.

**Figure 2:**
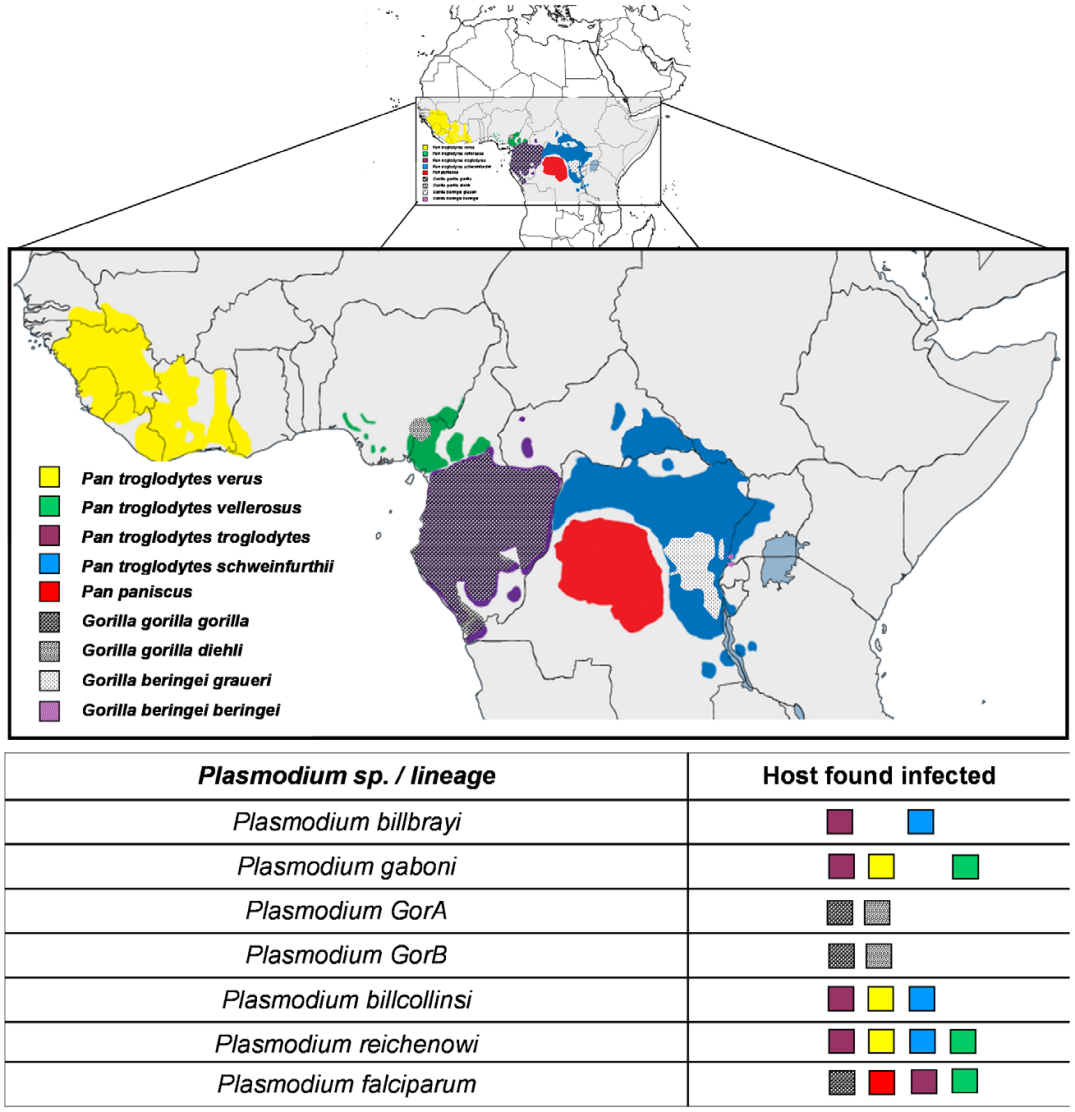
Distribution of the different subspecies of chimpanzees, bonobos, and gorillas in Africa and representation of the spread of the different *Plasmodium* species in these subspecies.

Thanks to the complementarity between the different studies, in terms of the geographic areas and host species and subspecies sampled, a clear picture of the partitions existing within the *Laverania* lineage now emerges. Figure 1 presents phylogenies of the *Laverania* subgenus obtained from partial *CytB* sequences extracted from several of the previously reported studies (methods used to construct the phylogeny are as those presented in Prugnolle et al. [22]; see also the Figure 1 legend for more details). Figure 2 represents the distribution of the different newly recognized *Plasmodium* lineages in the different subspecies of chimpanzees, gorillas, and bonobos, and Table 1 presents an historic overview of the different names given to these lineages.

**Table 1:** Historic overview of the molecular descriptions and of the names given to the different lineages (seven lineages) of the *Laverania* subgenus.

Lineages of the *Laverania* subgenus found in the african great apes	1			2	3	4	5	6	7
**Host species**	Chimpanzee	Bonobo	Gorilla	Chimpanzee	Chimpanzee	Gorilla	Chimpanzee	Chimpanzee	Gorilla
**First molecular characterization**	Duval et al. [24]	Krief et al. [23]	Prugnolle et al. [22]	Escalante and Ayala [5]	Rich et al. [1]	Prugnolle et al. [22]	Krief et al. [23]	Ollomo et al. [19]	Prugnolle et al. [22]
**Name given in the first study**	*P. falciparum*	*P. falciparum*	*P. falciparum*	*P. reichenowi*	*P. reichenowi*	*P. GorB*	*P. billbrayi*	*P. gaboni*	*P. GorA*
**Names given in subsequent studies**	*P. falciparum*	*P. falciparum*	Duval et al. [24]: *P. falciparum*Liu et al. [25]: *G1*	*P. reichenowi*Liu et al. [25]: *C1*	Krief et al. [23]: *P. billcollinsi*Duval et al. [24]: *P. billcollinsi*Liu et al. [25]: *C3*	Duval et al. [24]: *P. GorB*Liu et al. [25]: *G3*	Duval et al. [24]: *P. billbrayi*Liu et al. [25]: *C2*	Rich et al. [1]: *P. reichenowi*Prugnolle et al. [22]: *P. gaboni*Krief et al. [23]: *P. gaboni*Duval et al. [24]: *P. gaboni*Liu et al. [25]: *C2*	Liu et al. [25]: *G2*

The lineage number (1 to 7) is given following the phylogeny presented in Figure 1A.

As shown (Figure 1), the *Laverania* subgenus is subdivided in two main groups. We will refer to them as Group A and Group B. Group A is formed by two distinct and well-supported clades (Figure 1A and 1B). The first one is found in two gorilla subspecies, *Gorilla gorilla gorilla* and *G. g. dielhi* (Figure 2), and was originally described by Prugnolle et al. [22] as *P. GorA* (Table 1). The second clade is found in chimpanzees and is subdivided into two well-supported lineages (Figure 1A). The first includes the isolates called *P. gaboni* [19]; the other lineage is composed by *P. billbrayi* isolates [23] (Table 1). As shown in Figure 2, *P. gaboni* can infect at least three chimpanzee subspecies, *P. troglodytes verus*, *P. t. vellerosus*, and *P. t. troglodytes*, whereas *P. billbrayi* infects *P. t. troglodytes* and *P. t. schweinfurthii*.

**Figure 3:**
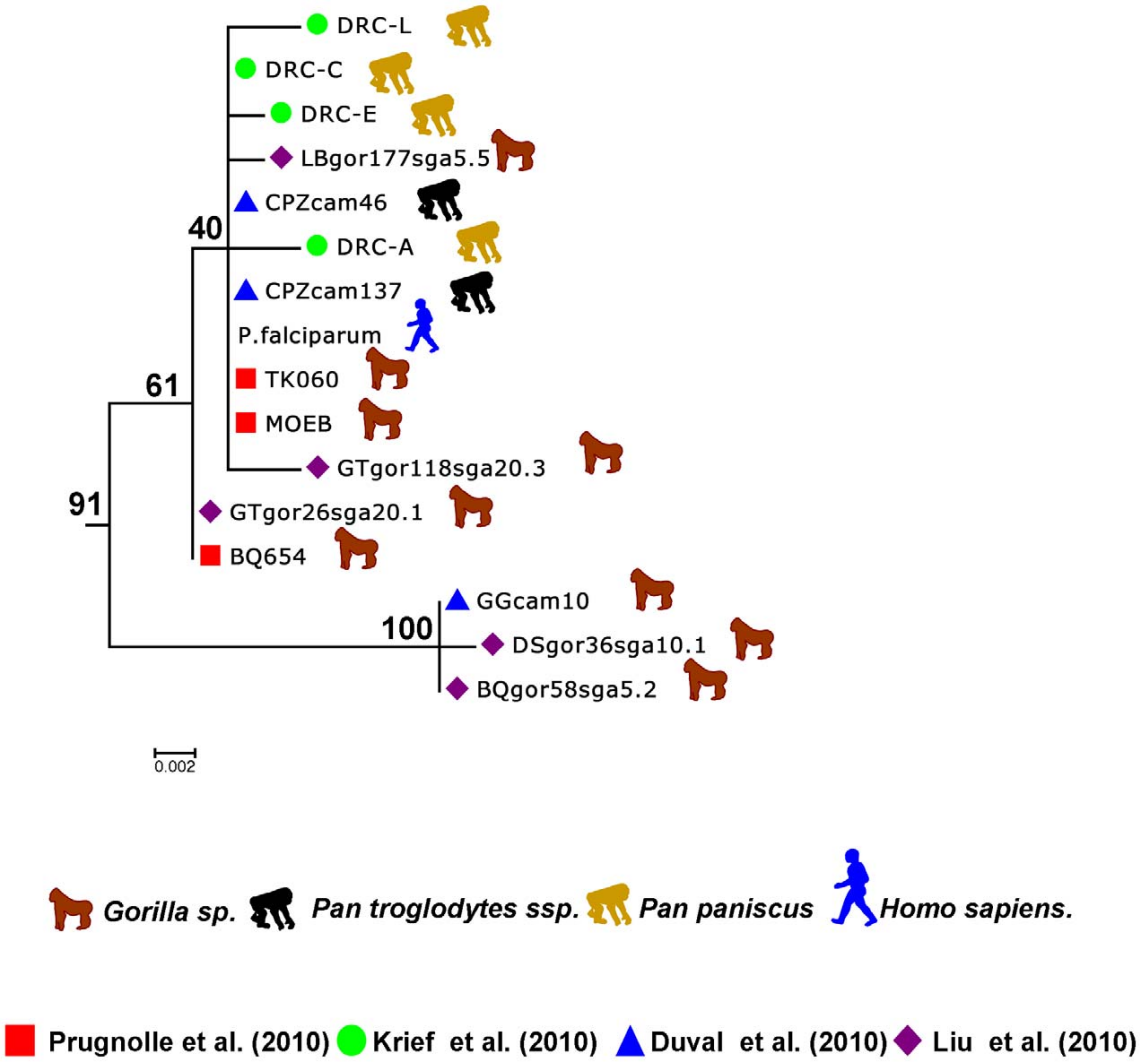
Sub-tree of the *P. falciparum* isolates. This sub-tree was extracted from the tree presented in Figure 1B and built using the data from Ollomo et al. [19], Prugnolle et al. [22], Krief et al. [23], Duval et al. [24], and Liu et al. [25].

Group B, the second main group of the *Laverania* clade, includes four distinct and well-supported lineages (Figure 1). The first is defined as *P. GorB* [22] (Table 1) and is constituted of isolates found in western lowland gorillas (subspecies *G. g. gorilla* and *G. g. dielhi*, Figures 1 and 2). The second main lineage, named *P. billcollinsi*, infects chimpanzees [23] (Table 1). As shown in Figure 2, to date, *P. billcollinsi* has been identified in three chimpanzee subspecies (*P. t. verus*, *P. t. troglodytes*, and *P. t. schweinfurthii*). The last two lineages are those of *P. reichenowi* and *P. falciparum*, respectively. As for the other *Plasmodium* species that infect chimpanzees, *P. reichenowi* is widespread and infects the four subspecies of chimpanzees (Figure 2). Until 2009, only one isolate (*P. reichenowi* CDC) was known, but now a lot of new isolates have been characterized in several different studies [21], [22], [24], [25]. Finally, as shown (Figures 1 and 3), gorillas [22], [25], bonobos [23], and chimpanzees [24] can also be infected by *P. falciparum*, although it used to be considered to be naturally strictly human specific.

### …and on the Origin of *P. falciparum*

During the last year and as briefly described above, the proposed scenarios concerning the origin of *P. falciparum* have changed several times (origin in chimpanzees, bonobos, or gorillas) depending on the host species analyzed, the lineages of *Plasmodium* discovered, or the way data were analyzed.

Now it seems sure, in light of all the studies, that *P. falciparum* did not originate from a transfer from birds, lizards, or rodents but instead derived from some *Plasmodium* lineages evolving in African hominids. More precisely, the study by Liu and collaborators [25] (see also Figure 3) strongly suggests that *P. falciparum* is of gorilla origin and that all known strains circulating in humans nowadays have resulted from a single cross-transmission event from gorilla to human. The origin of the *Laverania* subgenus in itself remains an enigma, but we will not further discuss this issue here.

Although the scenario of a *P. falcirparum* gorilla origin is very likely in light of the data recently presented [25], we think nevertheless that more information should be gathered before being able to definitely conclude that *P. falciparum* really originated in gorillas. First of all, it is still impossible to definitely rule out some alternative scenarios. Among them, one possibility could be that *P. falciparum* diverged from *P. reichenowi* at the time of the divergence between chimpanzees and humans (as recently suggested by Hughes and Verra [26]) and then was transferred several times from archaic humans to gorillas over the history of the lineage. The small genetic diversity observed within the current human *P. falciparum* isolates (compared to the one observed in gorillas) being the result of a recent and rapid expansion of only a small fraction of the human *P. falciparum* diversity, at the moment, for instance, of the expansion of modern humans from a small east African source population (as recently proposed by [27]).

Second, and despite the very large number of individuals that were analyzed by Liu et al. [25], there is still a possibility that *P. falciparum*–related strains circulate in the other African great apes (chimpanzees and bonobos, as shown by Duval et al. [24] and Krief et al. [23]) but at lower prevalence [28]. Moreover, given the propensity of the genus *Plasmodium* to switch from one host to another—as exemplified by the transfer of *P. knowlesi* from macaques to humans [29] and *P. vivax* between humans and South American monkeys [30]—it would not be very surprising to find *P. falciparum*–related strains or other *Laverania* lineages infecting the other non-human primates in Africa. New data should be gathered to confirm or infirm this possibility.

## Challenges for the Future

### Causes of the Host Switch

Explaining why the host switch from gorilla to human occurred in the evolutionary history of the *P. falciparum* lineage will certainly be one of the greatest challenges for the near future.

The process of emergence of a disease into a new host can be schematized by four transition stages according to [31]. The first two stages are prerequisites for the emergence itself: (1) a human contact with the infectious agent and (2) the cross-species transmission. The two others are necessary for the development of the pandemicity: (3) sustained human-to-human transmission and (4) genetic adaptation to humans.

Describing these transition stages, or at least getting a better picture of what happened at these different stages, will require the involvement of different scientific disciplines, from anthropology to entomology, immunology, and genetics. The identification as well as the description of the ecology and the biology (in particular the trophic behavior and host preference) of the vectors of the different *Laverania* species may, in our mind, constitute a good start to understand the origin of the *P. falciparum* host transfer.

### Dating the Events of Divergence

Dating the divergence time between the different species or lineages may help to understand the origin of *P. falciparum* and determine the factors that have led to speciation within the *Laverania* subgenus. For pathogens like those of the genus *Plasmodium*, speciation generally occurs through two main processes: 1) co-evolution with the hosts or 2) host switch. Estimating and comparing the *Plasmodium* divergence dates with those of the hosts should therefore allow to determine which process was involved. This implies, obviously, that the host phylogeny (be it the vertebrate or the vector phylogeny) has to be known or determined.

For parasites and soft organisms in general, fossil records are very rare if not absent. This implies that calibration of phylogenies is mostly based on speculation. The recent, aforementioned studies fell into this category. Ollomo et al. [19] proposed that *P. gaboni* diverged from the *P. reichenowi*/*P. falciparum* taxa about 21 MY ago based on the hypothesis that *P. reichenowi*/*P. falciparum* diverged between 4 and 7 MY ago. Krief et al. [23] considered other calibration points, as speculative as the one taken by Ollomo et al. [19]. They assumed, for example, that *P. gonderi* and macaque parasites co-diverged when *Macaca* branched from other *Papionina*, which led them to the conclusion that *P. falciparum* was transferred to humans from bonobos around 30,000 years ago.

In general, we advise readers to be cautious with time estimates and to give a second thought as to how these dates were obtained. In the absence of fossil calibration, one way to date the divergence between species is by using estimates of mutation rates [32]. The principle is that the time separating two sequences from a common ancestor (*T*) is a simple function of the substitution rate (*r*, which is equal to the mutation rate for neutral sites) and the observed divergence between sequences (*d*): *T = d/2r*. However, it is not easy to get good estimates of mutation rates. Ideally, they should be obtained for all the genes under study and for each species in the phylogeny because substitution rates may vary from one gene to another as well as among species [33]. Recently, Ricklefs and Outlaw [34] estimated the rate of substitution of *CytB* in *Plasmodium* species that infect birds and used this data to compute the divergence time of *P. falciparum* from *P. reichenowi*. They proposed a split around 2.5 MY ago, but the same previous remarks regarding the variation of substitution rates among unrelated lineages can be applied to this study.

Another possibility for calibrating a phylogeny is to use external calibration points such as well-documented events of vicariance caused by well-dated ancient geological or geographical events (e.g., [28]). In the case of *Plasmodium*, such an event could be, for example, the separation of Madagascar from the African continent (L. Duval, personal communication). Duval et al. [24] recently published the molecular description of a *Plasmodium* species isolated from a lemur of Madagascar (*Plasmodium malagasy*). Many studies have shown that the lemurs have been geographically isolated from other African primates for more than 55 MY [35], [36]. Because the island of Madagascar was always free from other non-human primate species, it should thus be possible to use this external calibration to date the other divergence events in the phylogeny.

### Genome Evolution

All these recent discoveries open up the possibility to thoroughly study the evolution of *Plasmodium* species and their genome. Sequencing the new species' genome should indeed allow the analysis of lineage-specific evolution using comparative genomics, and hence, the identification of the genes responsible for the adaptation of these parasites to their specific hosts [37]. Genome comparison will advance our understanding of the differences in malaria pathology and the processes at work in the interaction with the vertebrate or the mosquito hosts. It is thus essential to rapidly complete the sequencing of the phylogenetically important *Plasmodium* species within the *Laverania* lineage, in order to enhance our knowledge on the functional genomics of the most malignant human malaria parasites and of the genetic adaptation that might have facilitated its transfer from gorilla to human.

### Risk of Emergence: Human Invaded or Human Invader

The recent discovery of *P. falciparum* in bonobos [23], chimpanzees [24], and gorillas [22], [24] as well as *P. ovale*, *P. malariae*, and *P. vivax* in chimpanzees, bonobos, and gorillas [23], [24], [38], [39] highlights the risk of transfer of *Plasmodium* species from human to primates and vice versa. It is now urgent to identify the genetic and ecological factors that allow this group of pathogens to exploit a variety of host species. Notably, this feature should be of concern for the wildlife conservationist community, as recurrent release of human infectious diseases to great apes may accelerate their disappearance. Similarly, it is now important to systematically survey the presence of primate *Plasmodium* species in human populations, especially in those living in their vicinity (e.g., forest-dwelling populations) in order to evaluate if great apes may constitute a reservoir of *Plasmodium* for humans and the risk of emergence.

### Basic Biology of the *Laverania* Species

Finally, it will be essential to gather information on the biology and ecology of the different species belonging to the *Laverania* lineage and on their interactions with the hosts. We think in particular that it will be of major interest to investigate their virulence against chimpanzees and gorillas and thus determine if they impose selective pressures on them. Today, our knowledge on this aspect of their biology is still very limited (see however [23], [40]). In parallel, it will also be interesting to document the response to infection of the hosts and determine, in particular, if they have evolved mechanisms of resistance.

## Concluding Remarks

In conclusion, the recent data gathered from great apes in Africa have shown that a large diversity of *Plasmodium* species circulates among our relatives and have provided new insights into the evolutionary history of the malaria parasites of humans, particularly *P. falciparum*. These discoveries not only dramatically change our view on the evolution of the *P. falciparum* lineage, but also question the evolution and origin of the other human *Plasmodium* species (*P. ovale, P. malariae*, and *P. vivax*). This opens completely new areas of research and will certainly attract the attention of an all new community of scientists from various disciplines. Indeed, getting more information on the biology, ecology, and evolution of the different *Plasmodium* species infecting great apes will certainly help us to better understand and, therefore, fight against, the most virulent human malaria agent, *P. falciparum*.
